# Identification of Key Biomarkers for Systemic Lupus Erythematosus Progression and Therapy Response: A Bulk RNA‐Sequencing‐Based Bioinformatics Study

**DOI:** 10.1002/brb3.71098

**Published:** 2025-11-28

**Authors:** Luofei Huang, Quanzhi Lin, Han Li, Jian Shi

**Affiliations:** ^1^ Liuzhou Municipal Liutie Central Hospital Liuzhou Guangxi China; ^2^ Department of Internal Medicine The First Affiliated Hospital of Guangxi University of Science and Technology Liuzhou Guangxi China; ^3^ Department of Internal Medicine Liuzhou People's Hospital Liuzhou Guangxi China; ^4^ Department of Internal Medicine The People's Hospital of Laibin Laibin Guangxi China

**Keywords:** diagnostic biomarkers, diagnostic model, immune infiltration, molecular docking, systemic lupus erythematosus

## Abstract

**Background:**

Systemic lupus erythematosus (SLE) is a chronic autoimmune disease that affects multiple organs and has a higher prevalence in women of reproductive age. The increasing global incidence of this disease poses a significant public health challenge. Current diagnostic methods are delayed because of nonspecific clinical manifestations and the limited specificity of available tests. Furthermore, conventional treatments frequently exhibit considerable side effects and fail to achieve complete disease control in many patients. Therefore, identifying novel diagnostic biomarkers and treatment targets is imperative. This study integrated transcriptomics, protein interaction analysis, and immunological infiltration assays to identify and confirm key biomarkers in SLE.

**Methods:**

We obtained data from the Gene Expression Omnibus database. Weighted gene co‐expression network analysis (WGCNA) was used to identify gene modules, and differentially expressed genes (DEGs) were identified using limma software. Functional enrichment analyses were performed using gene ontology and the Kyoto encyclopedia of genes and genomes. Protein‐protein interaction (PPI) networks were generated using STRING and illustrated using Cytoscape. Machine learning algorithms, including the least absolute shrinkage and selection operator, random forest, and artificial neural networks (ANN), were employed to identify key diagnostic genes. A diagnostic model was constructed and verified, potential therapeutic compounds were predicted using the Drug Signature Database, and molecular docking was performed for drug‐target interaction analysis.

**Results:**

The WGCNA revealed a key gene module (red module) comprising SLE‐relevant genes. We identified 238 overlapping DEGs enriched in several immune‐related biological processes and signaling pathways. Fourteen common hub genes were identified from the PPI network. Four genes, SLC4A1, GATA1, DMTN, and SNCA, emerged as potential diagnostic biomarkers based on machine learning analysis. These genes were significantly correlated with immune cell infiltration patterns in SLE. A diagnostic ANN model that included these genes exhibited high predictive accuracy. A nomogram was constructed and validated. Drug prediction analysis identified N‐acetyl‐L‐cysteine as a viable treatment option, demonstrating a stable binding affinity in molecular docking simulations.

**Conclusions:**

This study demonstrates that SLC4A1, GATA1, DMTN, and SNCA are potential biomarkers for diagnosing SLE and monitoring therapeutic efficacy. The integrated diagnostic approach exhibited robust predictive power, and N‐acetyl‐L‐cysteine was identified as a viable therapeutic agent. These findings provide valuable insights for enhancing the early diagnosis and development of targeted therapies for SLE.

## Graphical abstract

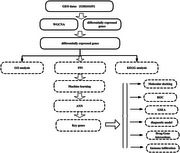

## 1 Introduction

Systemic lupus erythematosus (SLE) is a complex and chronic autoimmune disease characterized by the generation of autoantibodies and immune complex deposition, resulting in multi‐organ involvement and inflammation (Kiriakidou and Ching 2020; Zhou et al. 2024). In recent decades, the prevalence of SLE has increased, partly due to enhanced diagnostic techniques and increased awareness, rendering it a significant public health issue (Tian et al. 2023; Gergianaki et al. 2018). The chronic and relapsing nature of SLE severely impairs the quality of life of patients and imposes a substantial economic burden on healthcare systems and society (Ruiz‐Irastorza et al. 2001; O'Neill and Cervera 2010).

The current diagnosis of SLE primarily depends on a combination of clinical symptoms, laboratory investigations, including antinuclear antibody (ANA) detection, and histopathological examinations (Nashi and Shmerling 2022; Nashi and Shmerling 2021). However, these methods have some limitations. The clinical manifestations of SLE are highly heterogeneous, and some early symptoms may be nonspecific, resulting in diagnostic delays (Gill et al. 2003). Although ANA is a common screening test, it lacks sufficient specificity, frequently resulting in false positives for various autoimmune and non‐autoimmune diseases (Kwon et al. 2020). Furthermore, histopathological examination is invasive and not consistently practical for assessing early‐stage diseases. Although conventional strategies, including corticosteroids and immunosuppressive drugs, are somewhat effective, they are associated with significant side effects, such as infections, osteoporosis, and metabolic disorders (Durcan et al. 2019’ Kuhn et al. 2015). The emergence of biologics, including belimumab, has provided new hope for treating this condition. However, numerous patients continue to experience incomplete disease management and persistent exacerbations (Singh et al. 2021).

Given the ongoing challenges in SLE diagnosis and treatment, the identification of new and reliable diagnostic biomarkers is urgently required. These biomarkers could enhance diagnostic accuracy and timeliness while offering essential insights into the molecular mechanisms underlying disease pathogenesis. Therefore, understanding the relationship between these diagnostic markers and the biological pathways driving SLE may facilitate new therapeutic interventions, enabling the development of more effective and targeted treatment strategies for patients (Yu et al. 2021; Harden and Hammad 2020). To achieve this goal, we conducted a comprehensive analysis of a large‐scale cohort of patients with SLE. We aimed to identify key diagnostic markers of SLE by integrating advanced bioinformatics techniques, high‐throughput sequencing, and machine learning algorithms. Our primary aim was to investigate the potential for developing new therapeutic strategies based on these markers, which could transform the existing management of SLE and enhance patient prognosis.

## 2 Research Approach

### 2.1 Data Source

**FIGURE 1:**
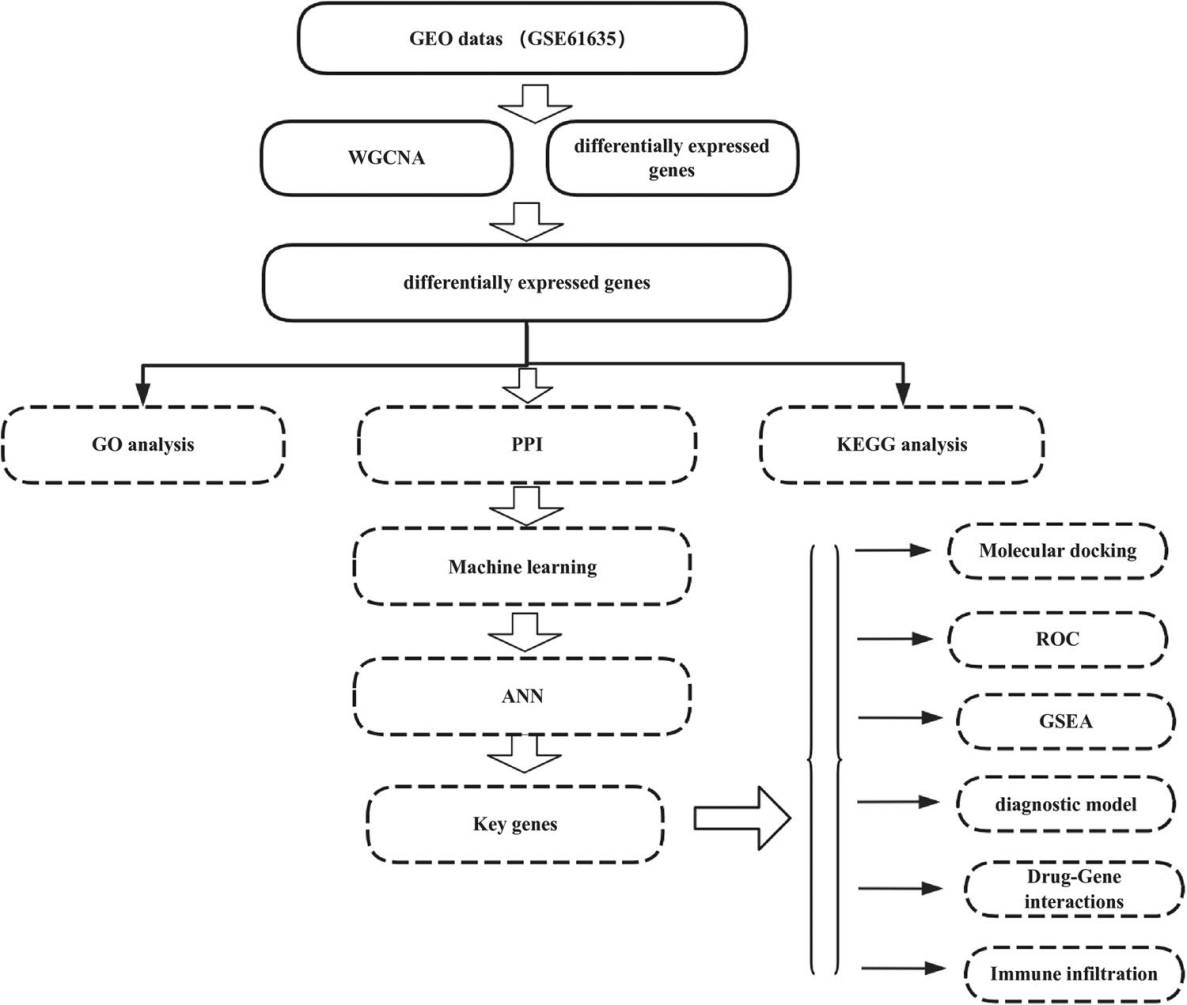
The study flowchart.

All datasets analyzed were obtained from the Gene Expression Omnibus (GEO) database (https://www.ncbi.nlm.nih.gov/geo/) (Barrett et al. 2013). The GSE61635 dataset was used to examine mRNA in blood samples from a cohort of 79 patients with SLE, including those who underwent follow‐up visits. This resulted in 99 arrays and 30 healthy participants, with one array per participant. Figure 1 depicts the analytical workflow of this study.

### 2.2 Weighted Gene Co‐Expression Network Analysis (WGCNA)

This study utilized the WGCNA package in R software to explore gene modules closely associated with the clinical characteristics of the disease (Langfelder and Horvath 2008). Previous studies (Xu et al. 2024) indicated that hierarchical clustering was initially employed to identify and eliminate abnormal outlier samples based on the following principles: First, a gene expression matrix was used to quantify expression differences between samples via Euclidean distance. Next, the method used average linkage to iteratively merge the closest samples or clusters. This process generated a dendrogram that reflects the similarity of the expression patterns. Outliers were identified using a static cutoff height of 20,000 in the dendrogram. A minimum cluster size of 10 branches exceeding the height threshold and containing fewer than 10 samples was used to classify them as outliers. Validation was conducted through consistency checks against the clinical traits to ensure reliability. Subsequently, the “Pick Soft Threshold” function was used to iteratively evaluate soft‐threshold power vectors (powerVector = 1–20). The optimal parameter *β* = 12 was determined using two criteria. First, it met the scale‐free topology model fit criterion (*R* ^2^ ≥ 0.9). Second, it maintained a consistent mean connectivity, achieving an *R* ^2^ of 0.91 and stabilized connectivity indicative of biologically relevant module structures. A gene adjacency matrix was created and transformed into a topological overlap matrix (TOM), which underwent average‐linkage hierarchical clustering to group genes with similar expression patterns based on TOM metrics. Finally, the correlation coefficients between each gene module and clinical traits were calculated to quantify their associations (Pei et al. 2017).

### 2.3 Identification of Differentially Expressed Genes (DEGs)

Differential expression analysis between the control and experimental groups was performed using the R language (Liu and Li 2022; Li and Liu 2022). Consistent with previous studies, the analyses were primarily conducted using the Limma software. First, the gene expression matrix and sample grouping files were read, followed by data preprocessing, including the merging of duplicate genes and inter‐sample standardization. Subsequently, grouping comparison relationships were defined using design and contrast matrices. After model fitting, significant DEGs were filtered based on the criteria of *p* < 0.05 and absolute log2 fold change (FC) ≥ 0.585. Finally, tools such as pheatmap and ggplot2 were used to generate heatmaps and volcano plots of DEGs.

### 2.4 Functional Enrichment Analysis

We performed gene ontology (GO) analysis, encompassing biological processes, cellular components, and molecular functions (Chen et al. 2017), along with Kyoto encyclopedia of genes and genomes (KEGG) pathway enrichment analysis (Kanehisa and Goto 2000), as in previous studies (Liu and Weng 2022; Liu and Weng 2022). These analyses were performed using the ClusterProfiler package in R software within the framework of the disease ontology. A gene set enrichment analysis (GSEA) tool was used to investigate pathway enrichment. The gene set c2.cp.kegg.v7.3. symbols obtained from the official ggt website served as a reference during the analysis. A statistical significance threshold of *p* < 0.05 was established for all enrichment analyses (Subramanian et al. 2005).

### 2.5 Protein‐Protein Interaction (PPI) Network Analysis

We constructed PPI networks for the identified genes using the STRING tool (version 11.5) with a medium confidence threshold (combined score ≥ 0.4) as the screening criterion and illustrated the networks using Cytoscape (Wang et al. 2020), consistent with the methodologies used in previous studies (Ou et al. 2024; Liu et al. 2023). Within the constructed PPI networks, we used the Molecular Complex Detection (MCODE) plugin in Cytoscape to identify highly interconnected modules. MCODE automatically detects densely connected regions based on node weighting and local neighborhood density, thereby revealing potential biological functional units or synergistic modules. The analysis was performed with the following parameters: node score cutoff of ≥ 0.2, k‐core of 2, and maximum depth of 100, to screen for biologically significant subnetworks.

### 2.6 Machine Learning‐Based Biomarker Screening and Predictive Performance Evaluation

We employed the least absolute shrinkage and selection operator (LASSO) regression model implemented using the glmnet package in R software, as indicated in previous studies for machine learning analyses (Liu et al. 2024; Liu and Tang 2023). The dataset was preprocessed by transposing the gene expression matrix and extracting sample labels using regular expressions (Wang et al. 2022). The model was subjected to 10‐fold cross‐validation (cv.glmnet) with family = “binomial” for binary classification, selecting the optimal penalty parameter *λ* through minimum deviance (lambda.min). A LASSO path plot was generated to illustrate coefficient shrinkage across *λ* values, with significant genes manually labeled at the final *λ* value. Concurrently, a random forest (RF) model was developed using the randomForest package. It was initially trained with 500 trees and optimized through the minimum out‐of‐bag error rate to ascertain the optimal tree count (bestTreeCount). Gene importance was assessed using the Gini index (MeanDecreaseGini), leading to the selection of the top 10 genes for further analyses. A bubble plot was then generated using ggplot2 to illustrate the scores arranged by descending Gini values (Jin et al. 2023). Additionally, a three‐layer artificial neural network (ANN) model with five hidden neurons was built using the neuralnet software. This dynamically generates the model formula based on DEGs and creates binary labels through median‐threshold binarization of expression data. The network's predictions were validated using confusion matrices to calculate class‐specific accuracy, while plot (nn_model) was used to display its architecture and weights, illustrating the connections between input genes, hidden layers, and output labels. All analyses ensured reproducibility using fixed random seeds (set.seed), data partitioning, and cross‐validation, while the results were saved to text files and visualizations were exported as PDFs. For data partitioning, the dataset was randomly divided into training (70%) and validation (30%) sets using stratified random sampling through the sample function in R, with the random seed fixed at 123 (set.seed(123)) to ensure reproducibility. The grouping strategy maintained the original distribution of patients with SLE and healthy controls in both the training and validation subsets. Sample labels were automatically extracted from the file names, with “1” assigned to SLE cases and “0” to healthy controls based on keywords, including “tre” and “con” in the sample IDs. This approach ensured the independence of the validation set for accurately evaluating the generalization ability of the ANN model. These machine learning approaches, including LASSO, RF, and ANN, were utilized to screen key diagnostic biomarkers, with receiver operating characteristic (ROC) curves evaluating their predictive robustness and identifying clinically significant genes as potential drug targets.

### 2.7 Immune Infiltration

CIBERSORT uses a gene expression‐based deconvolution algorithm to accurately measure the relative abundance of 22 subtypes of invading lymphocytes in each sample group (Chen et al. 2018). Subsequently, Pearson's correlation analysis was performed to comprehensively investigate the potential associations between key genes and immune cell subtypes.

### 2.8 Construction and Validation of the Diagnostic Model

We constructed a diagnostic model to predict the risk of SLE based on the identified key genes. The model was created using a dynamic nomogram and decision curve, and calibration and clinical effect curves were used to evaluate its accuracy. We generated ROC curves using R software to further validate the predictive performance of the model.

### 2.9 Drug‐Gene Interactions and Protein‐Protein Docking Analyses

This study integrated two complementary approaches to identify potential therapeutic agents and elucidate their molecular mechanisms of action. First, the Drug Signatures Database (DSigDB) was used to predict potential medications by linking the identified hub genes to their corresponding pharmaceutical compounds, capitalizing on DSigDB's comprehensive curation of drug‐gene relationships (Yoo et al. 2015). Subsequently, molecular docking simulations were performed to investigate the interactions between predicted drug candidates and target proteins. The chemical structures of the drug candidates were retrieved from the PubChem database, which offers comprehensive information on the molecular characteristics and 3D conformations of compounds (Wang et al. 2017). The 3D structures of the target proteins were obtained from the Research Collaboratory for Structural Bioinformatics (RCSB) Protein Data Bank, ensuring the use of high‐quality, experimentally verified models. The CB‐Dock2 platform, an advanced online tool for protein‐ligand docking, was used to perform docking simulations. It automatically identifies the binding sites and predicts the most favorable binding poses (Ou et al. 2024). The resultant docking data, including binding affinities and interaction details, were carefully analyzed to evaluate the stability and potential therapeutic efficacy of the identified drug candidates, thereby offering insights into their potential applications in treatment strategies.

## 3 Results

### 3.1 WGCNA‐Based Module Identification

**FIGURE 2:**
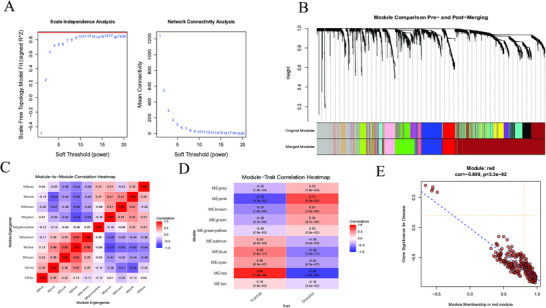
Identification of module genes. (A) A soft‐thresholding power analysis was employed to obtain the scale‐free fit index of the network topology. (B) Gene‐clustering dendrogram. (C) Heatmap depicting the associations between modules. (D) Heatmap illustrating the relationships between the modules and traits. (E) Correlation chart between gene members of the red module and gene significance.

In this study, WGCNA was used to identify the essential gene modules. After a comprehensive assessment, the soft‐thresholding power *β* was empirically set at 12 (Figure 2A). Subsequently, the dynamic tree cut algorithm was employed to partition the genes, yielding ten different gene modules (Figure 2B). Figure 2C illustrates the inter‐module relationship. Among these modules, only the red module exhibited significant correlations (Figure 2D). Correlation analysis revealed a robust negative association between the red module and gene significance, with a correlation coefficient of −0.8999 (*p* = 3.3 × 10^−92^, Figure 2E). Based on these findings, the red module was identified as the key module of interest, comprising 254 genes (Supplementary Table S1).

### 3.2 Identifying the DEGs

**FIGURE 3:**
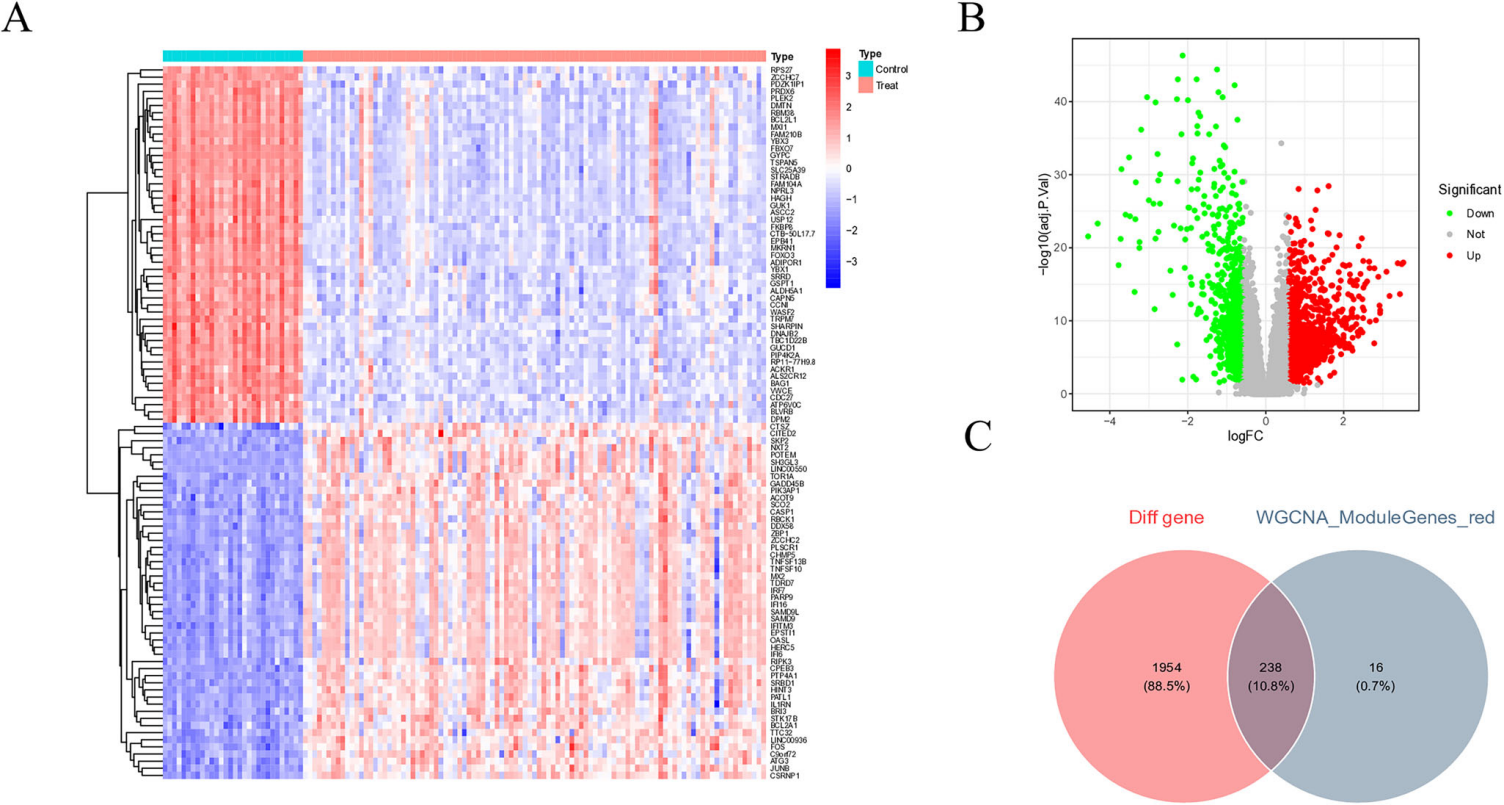
Identification of SLE‐associated DEGs. (A) Volcano plot of DEGs. (B) Heatmap of DEGs. (C) Venn diagram of related DEGs. SLE, systemic lupus erythematosus.

The expression profile dataset GSE61635 was subjected to normalization procedures. A volcano plot (Figure 3A) and a heatmap (Figure 3B) were generated to illustrate the data. We identified 238 DEGs that overlapped with the module genes through the analysis presented in Figure 3C.

### 3.3 Functional Enrichment Analysis of Intersection Genes

**FIGURE 4:**
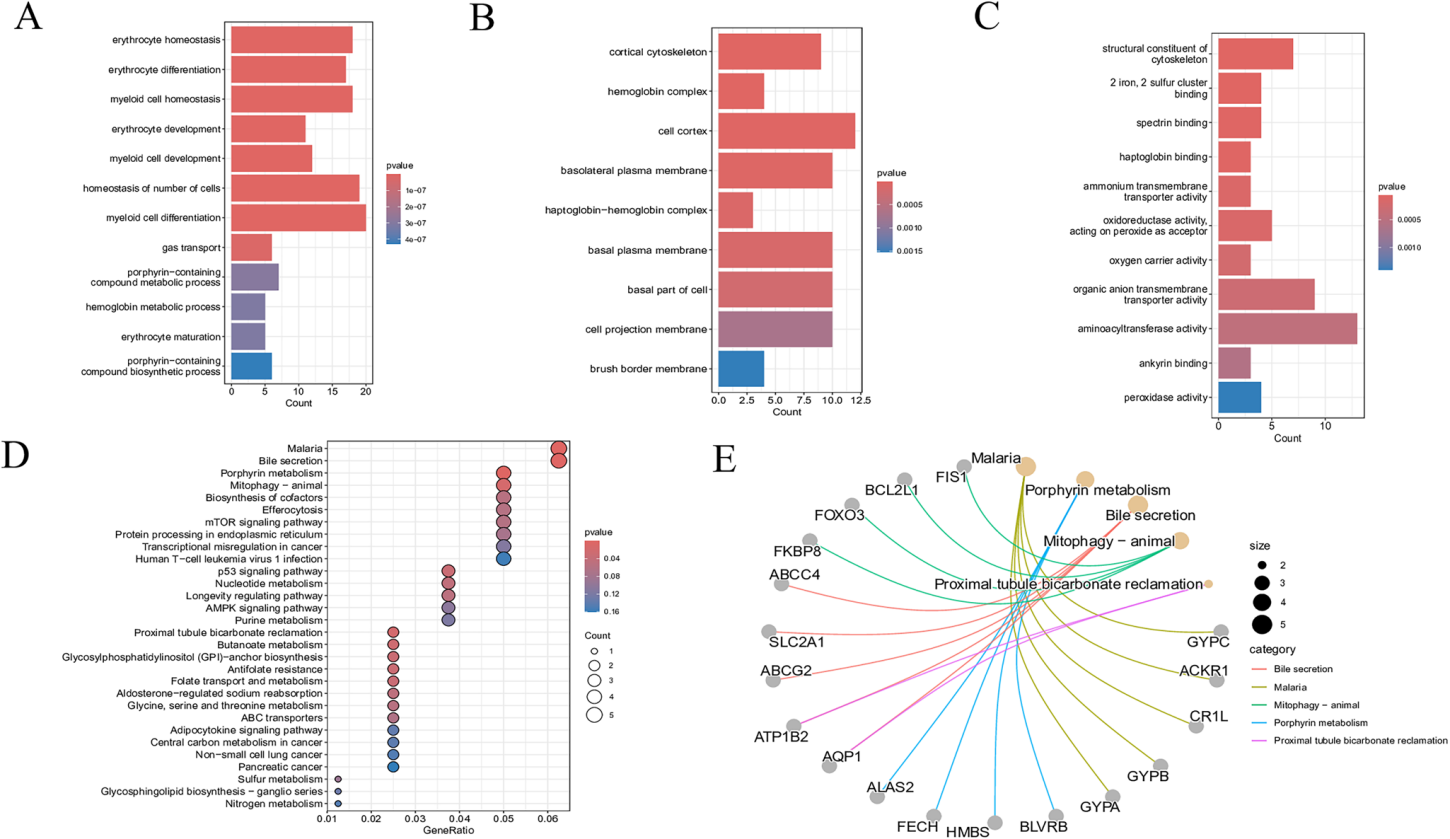
Functional enrichment analysis of DEGs. (A) GO biological processes. (B) GO cellular components. (C) GO molecular functions. (D) Bubble plot of KEGG pathways. (E) Interaction diagram of KEGG pathways.

Regarding the biological processes, Figure 4A depicts that the intersecting genes predominantly participate in cellular number homeostasis, myeloid cell differentiation, and myeloid cell homeostasis. Figure 4B displays that these genes primarily participate in the cell cortex and basolateral plasma membrane concerning cellular components. Figure 4C illustrates their association with functions, including acting as a structural component of the cytoskeleton, demonstrating aminoacyltransferase activity, and binding to two iron and two sulfur clusters.​ Furthermore, pathway enrichment analysis revealed that these genes were predominantly associated with malaria, bile secretion, and porphyrin metabolism pathways (Figures 4D‐E).

### 3.4 PPI Network and Hub Gene Analysis

**FIGURE 5:**
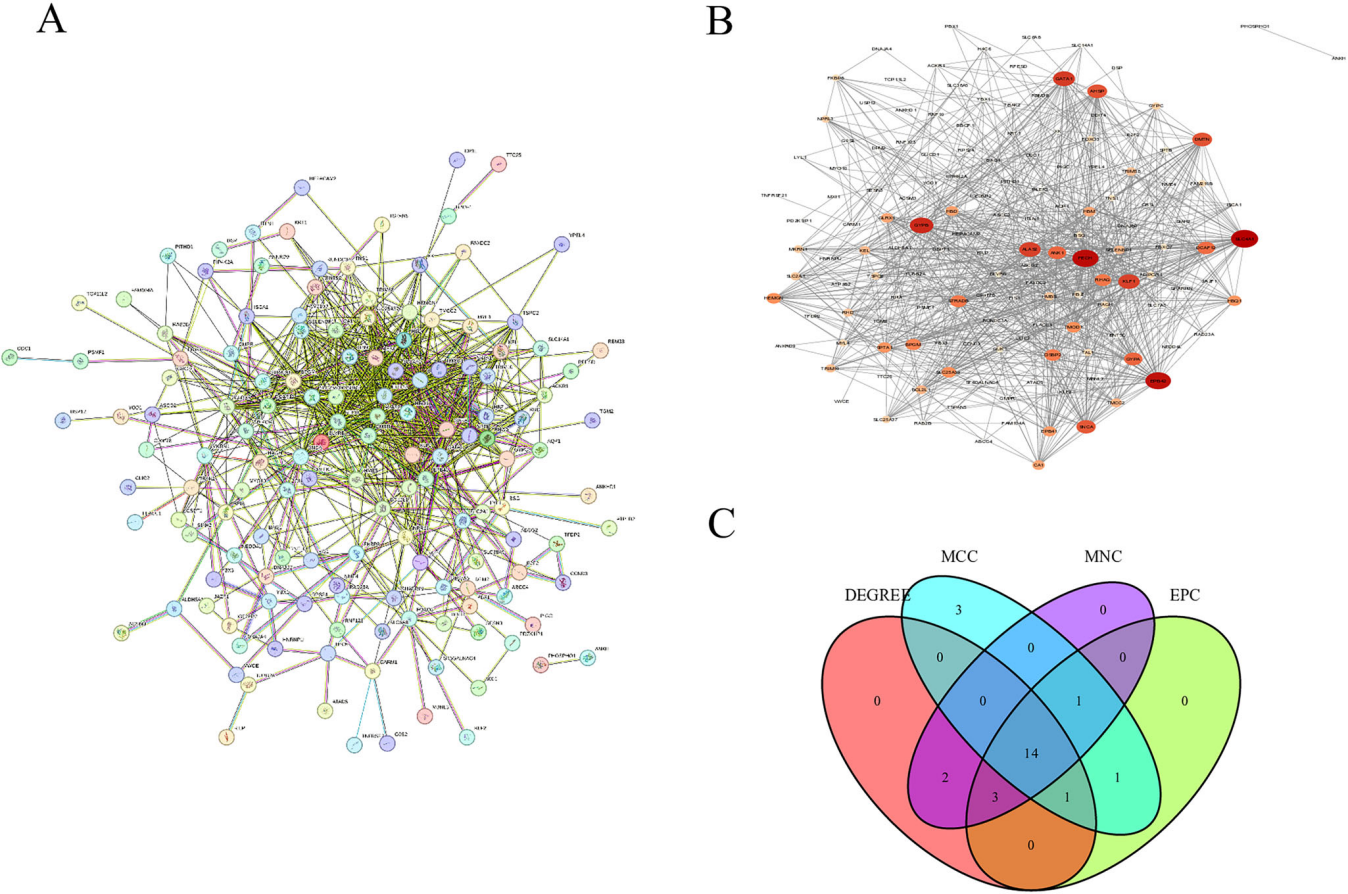
PPI network and hub genes analysis. (A) The PPI network of the DEGs, where larger edge sizes indicate a higher degree. (B) The first module of the PPI network. (C) We identified 14 common hub genes using four algorithms of the cytoHubba plugin.

Data were retrieved from the STRING database, and a PPI network was developed using genes from key modules identified by WGCNA. The resulting network comprised 178 nodes and 768 edges (Figure 5A). We used the MCODE plugin to identify densely connected regions within the network (Figure 5B). Four different algorithms from the cytoHubba plugin were used to rank and identify the top 20 hub genes. A comprehensive bioinformatics analysis identified 14 common hub genes: SLC4A1, FECH, EPB42, GYPB, GATA1, KLF1, ALAS2, DMTN, AHSP, SNCA, GYPA, ANK1, RHAG, and TMOD1 (Figure 5C).

### 3.5 Identification and Validation of the Key Genes

**FIGURE 6:**
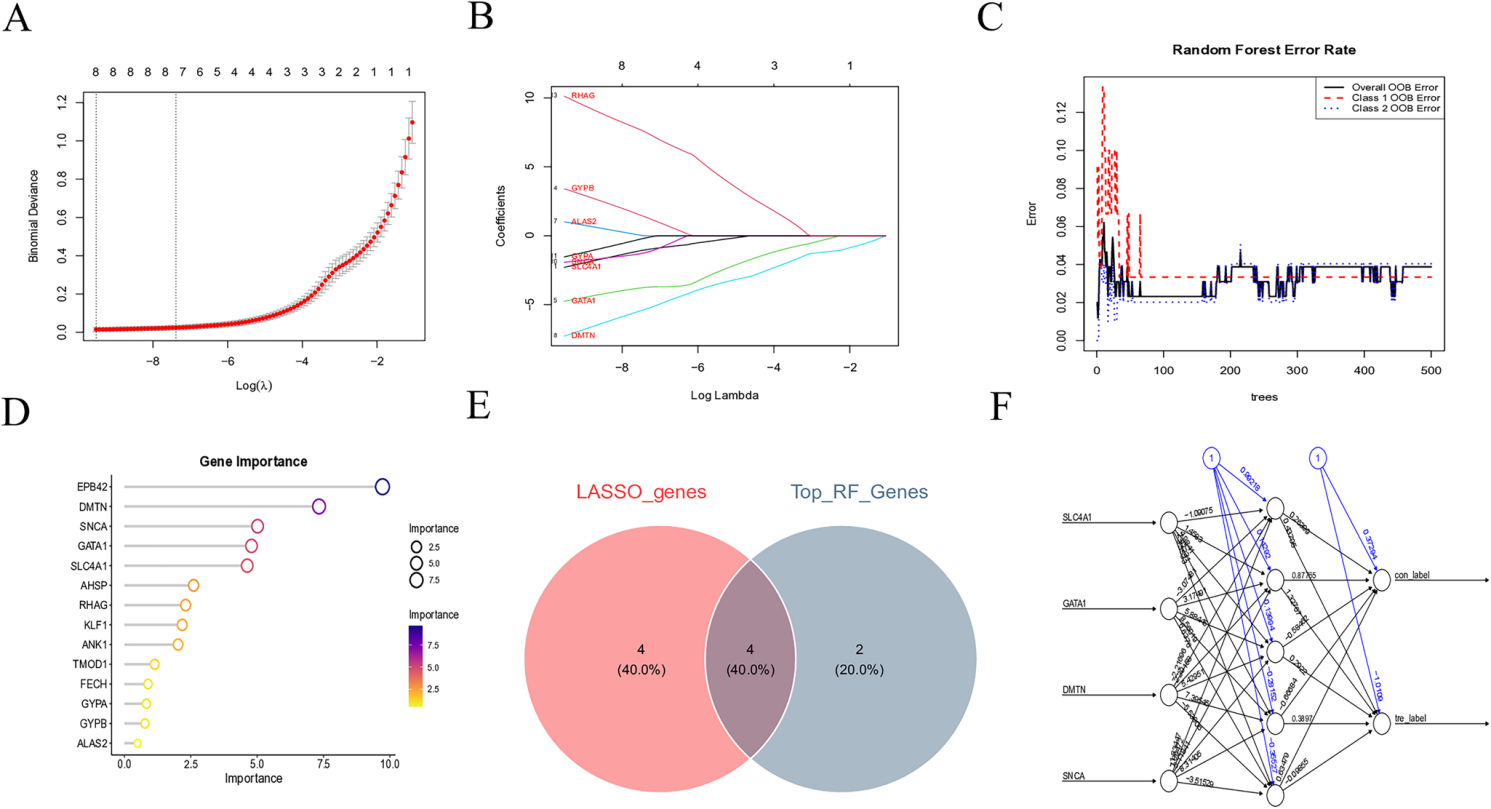
Identification of the key genes. (A‐B) Key genes were identified from hub genes using the machine learning LASSO regression method. (C‐D) Key genes were identified from hub genes using the RF algorithm. (E) Four key genes were identified through overlap analysis: SLC4A1, GATA1, DMTN, and SNCA. (F) The ANN model of the key genes.

**FIGURE 7:**
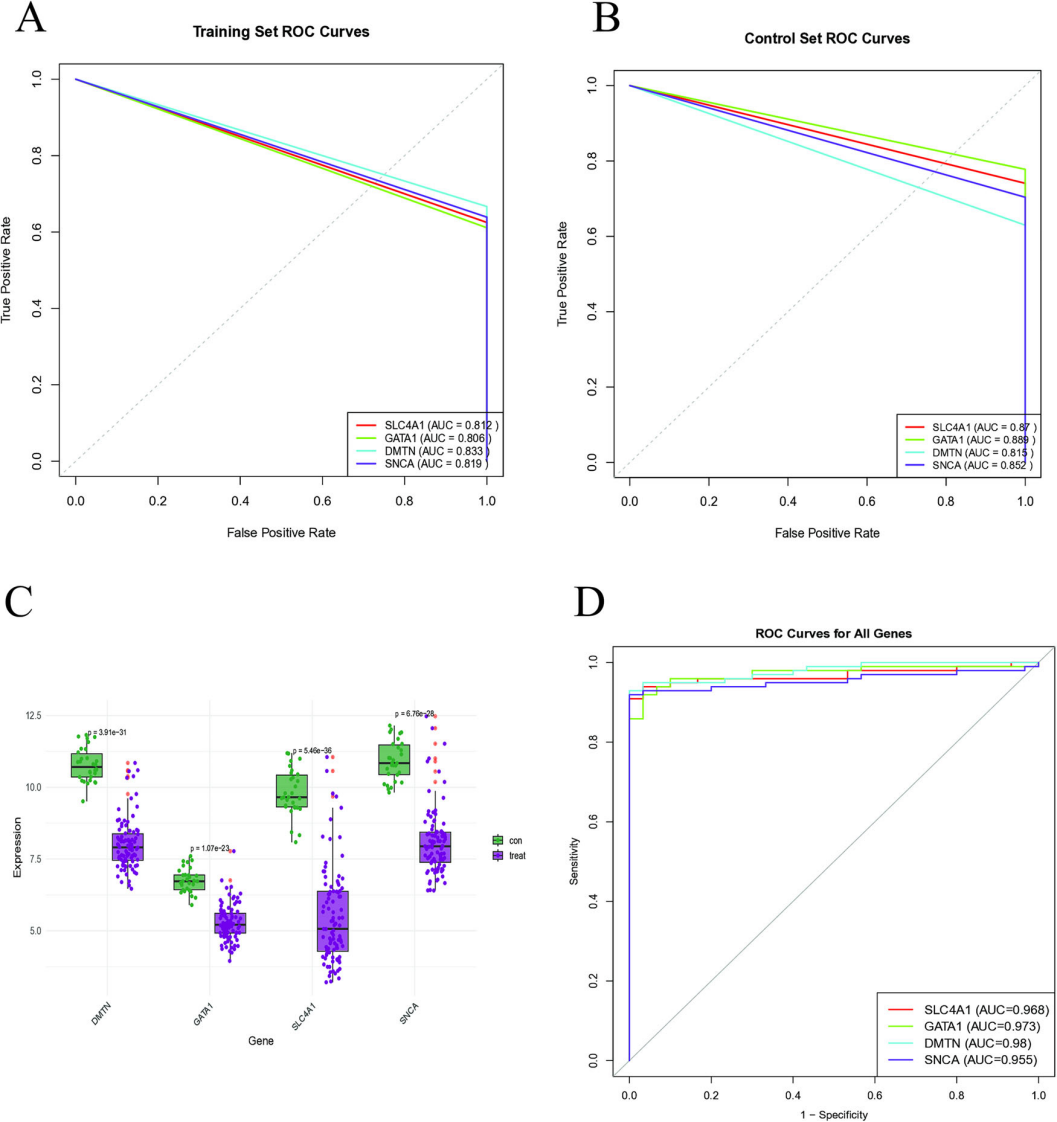
Validation of key genes. (A) ROC analysis of key genes in the experimental group of the ANN model. ​(B) ROC analysis of key genes in the validation group of the ANN model. (C) Key genes were significantly downregulated in SLE. (D) ROC curves of key genes in SLE. SLE, systemic lupus erythematosus.

We applied multiple machine learning algorithms to our analytical pipeline to identify reliable and pivotal biomarkers. The LASSO regression model identified eight candidate diagnostic biomarkers (Figures 6A‐B), whereas the RF algorithm selected ten genes with significant diagnostic potential (Figures 6C‐D). A Venn diagram comparison revealed four overlapping genes—solute carrier family 4 member 1 (SLC4A1), GATA binding protein 1 (GATA1), dematin actin binding protein (DMTN), and synuclein alpha (SNCA)—as significant diagnostic biomarkers (Figure 6E). Subsequently, an ANN model was constructed using the relative weights of these four genes (Figure 6F). The diagnostic performance of the model was evaluated using ROC curve analyses. The area under the curve (AUC) values for SLC4A1, GATA1, DMTN, and SNCA were 0.812, 0.806, 0.833, and 0.819, respectively (Figure 7A), indicating excellent predictive ability. These biomarkers maintained strong diagnostic performance in the validation cohort, with every AUC exceeding 0.7 (Figure 7B).

**FIGURE 8:**
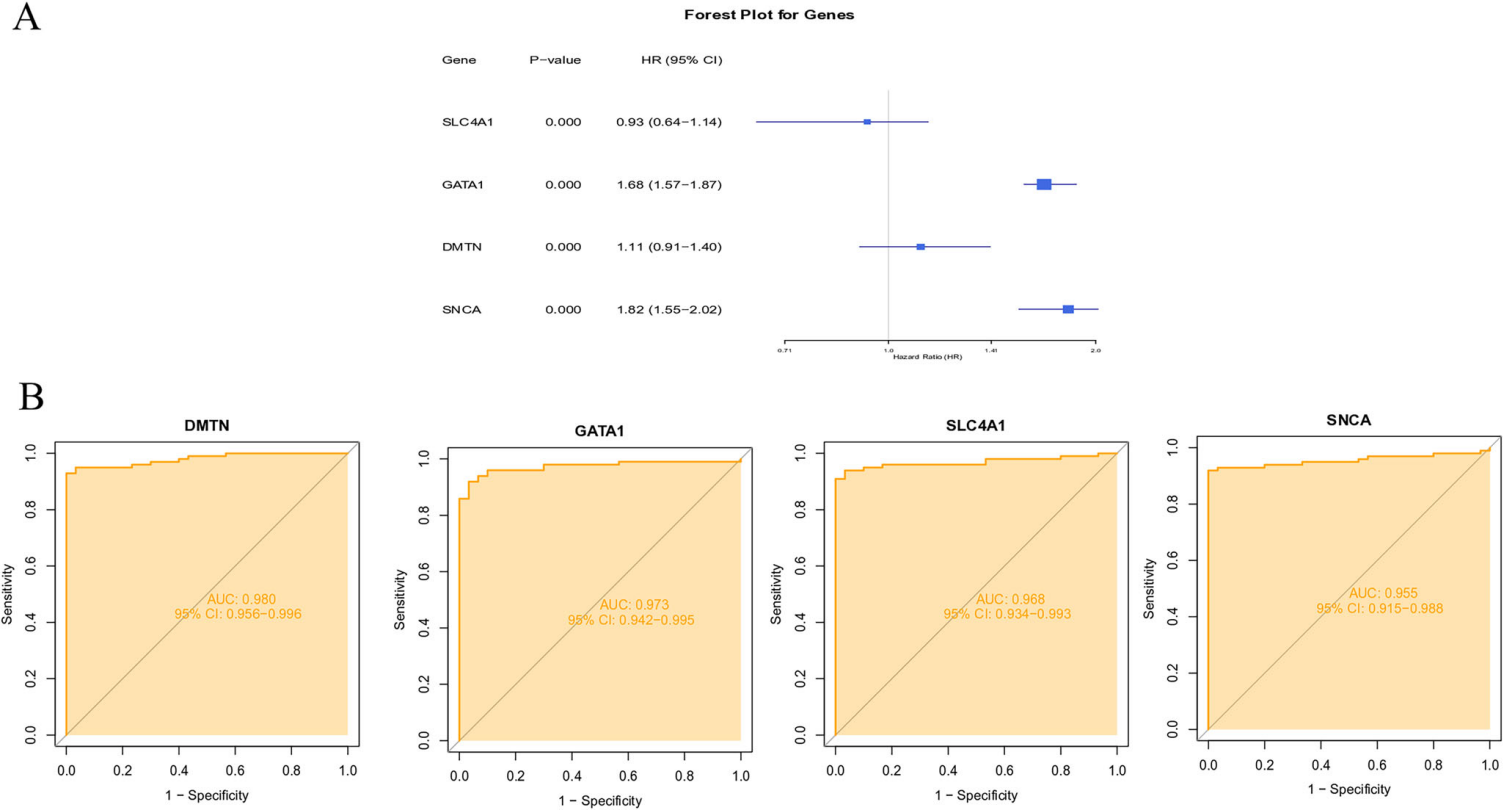
Visualization of key genes. (A) Forest plot of key genes in SLE. (B) ROC curves of key genes. SLE, systemic lupus erythematosus.

We also examined the clinical relevance of these four genes in patients with SLE. The expression levels of all four biomarkers were significantly different between patients with SLE and healthy controls (Figures 7C‐D). Forest plot analysis revealed that GATA1, DMTN, and SNCA were statistically significant risk factors for SLE, whereas SLC4A1 functioned as a protective factor (Figure 8A). Each gene had an AUC > 0.9, indicating strong diagnostic utility (Figure 8B).

### 3.6 Immune Infiltration and Enrichment Analysis

**FIGURE 9:**
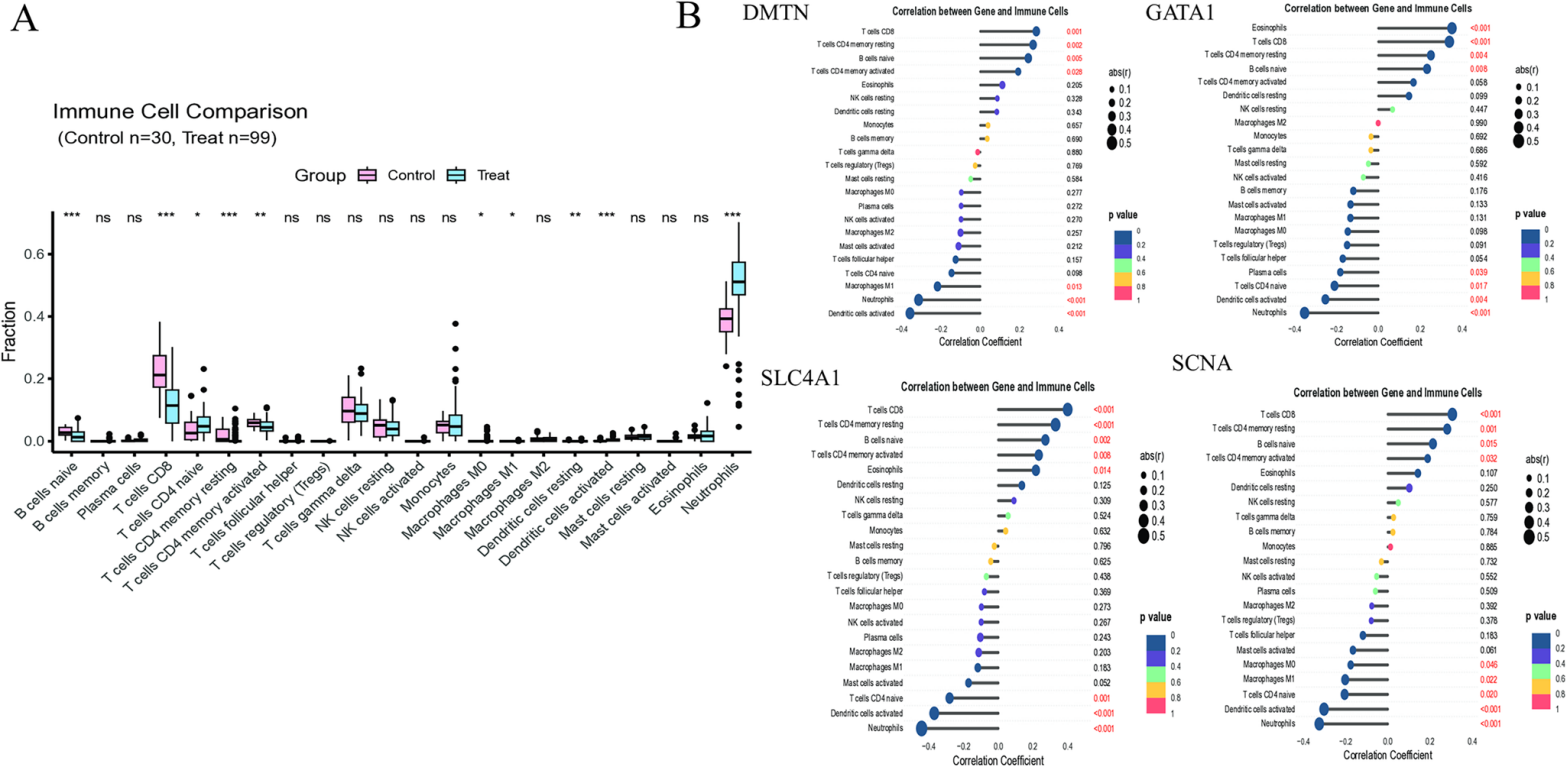
Immune infiltration and single‐cell analysis. (A) Proportions of immune cells in patients with SLE and the control group. (B) Correlation between key genes and immune cells in patients with SLE. (C) Expression levels of key genes in different cell types of patients with SLE and the control group. SLE, systemic lupus erythematosus.

The CIBERSORT algorithm was employed to investigate the immune infiltration landscape in SLE. A comparative analysis revealed that immune cell infiltration, particularly that of CD8⁺ T and activated memory CD4⁺ T cells, was significantly more elevated in the SLE group than in the healthy control group (Figure 9A). Correlation analysis revealed that the key gene GATA1 was significantly positively correlated with CD8⁺ T and resting memory CD4⁺ T cells. DMTN was positively associated with eosinophils and CD8⁺ T cells, whereas SLC4A1 was significantly positively correlated with CD8⁺ T and resting memory CD4⁺ T cells. Notably, SNCA exhibited a significant positive correlation with CD8⁺ T and resting memory CD4⁺ T cells (Figure 9B).

**FIGURE 10:**
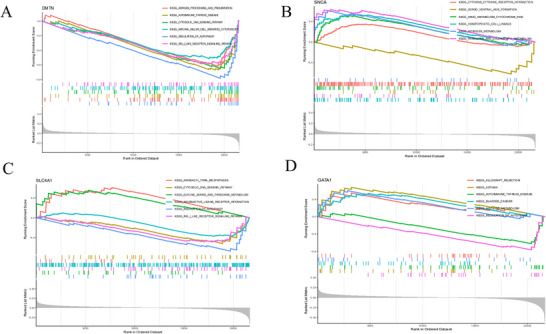
Gene set enrichment analysis. (A) GSEA analysis of DMTN. (B) GSEA analysis of SNCA. (C) GSEA analysis of SLC4A1. (D) GSEA analysis of GATA1.

GSEA revealed unique pathway enrichment patterns linked to the expression levels of principal genes. Low DMTN expression was enriched in immune‐related pathways, including antigen processing and presentation, autoimmune thyroid disease, cytosolic DNA sensing, natural killer cell‐mediated cytotoxicity, autophagy control, and RIG‐I‐like receptor signaling (Figure 10A). High SNCA expression was associated with pathways related to drug metabolism via cytochrome P450, hematopoietic cell lineage, nitrogen metabolism, and porphyrin and chlorophyll metabolism (Figure 10B). High SLC4A1 expression was primarily enriched in the aminoacyl‐tRNA biosynthesis and glycine, serine, and threonine metabolism pathways (Figure 10C). High GATA1 expression was associated with pathways related to allograft rejection and asthma (Figure 10D).

### 3.7 Development of an SLE Diagnostic Model Based on Key Genes

**FIGURE 11:**
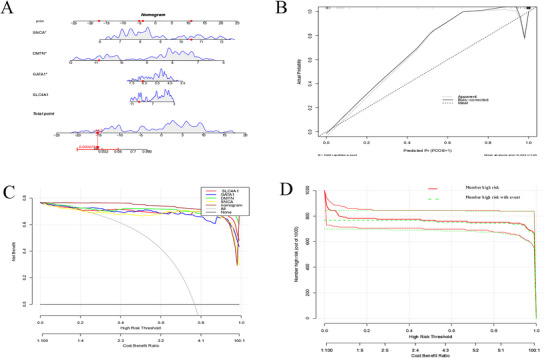
Construction and validation of the diagnostic model for SLE. (A) Nomogram for SLE. (B) Calibration curve of the model. (C) Decision curve analysis. (D) Clinical impact curve. SLE, systemic lupus erythematosus.

A nomogram model for SLE diagnosis was developed using the key genes SLC4A1, GATA1, DMTN, and SNCA. The dynamic nomogram (Figure 11A), calibration curve (Figure 11B), and decision curve analysis (Figure 11C) were generated using the R software. The calibration curve demonstrated a strong correlation between the SLE diagnostic model and the actual outcomes. Notably, within the 0.2–1 high‐risk threshold range, the “number high risk” curve closely matched the “number high risk with event” curve. This indicates the excellent predictive ability of the nomogram model (Figure 11D).

### 3.8 Candidate Drug Prediction

**FIGURE 12:**
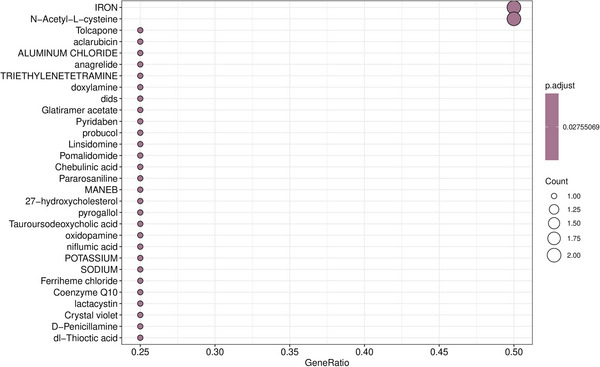
Visualization of drug enrichment of key genes.

Leveraging the DSigDB, we systematically predicted potential therapeutic interventions for SLE. Through our comprehensive analysis, several compounds demonstrating significant associations with SLE treatment were identified, and adjusted *p*‐values < 0.05 served as the criterion for statistical significance. Among these, N‐acetyl‐L‐cysteine and iron emerged as the most statistically robust candidates (Figure 12). Given the limitations of the available data, our comprehensive analysis primarily focused on N‐acetyl‐L‐cysteine. These findings underscore the promising potential of these identified compounds as viable therapeutic agents for SLE, indicating new directions for future research and clinical applications.

### 3.9 Molecular Docking

**FIGURE 13:**
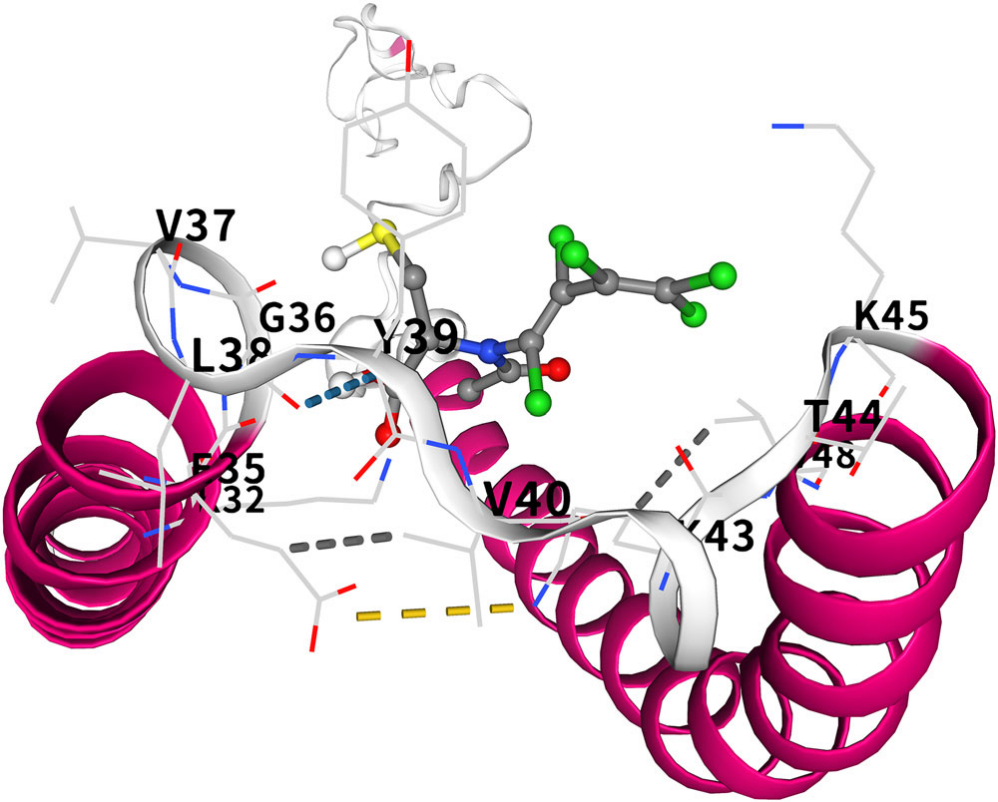
Molecular docking results of N‐acetyl‐L‐cysteine with SNCA protein.

To assess the therapeutic potential of the candidate medications, we performed molecular docking simulations to evaluate their binding affinities with the target proteins. Regarding N‐acetyl‐L‐cysteine, stable interactions were observed between crucial SNCA proteins. Notably, it exhibited a relatively low binding energy of –4.6 kcal/mol, indicating the formation of a highly stable protein‐ligand complex (Figure 13). These findings indicate that N‐acetyl‐L‐cysteine may serve as a basis for effective SLE treatment, offering a novel and promising avenue for future therapeutic development.

## 4 Discussion

This study performed a comprehensive analysis of SLE‐associated molecular mechanisms and identified potential diagnostic and therapeutic targets (Mills 1994). By integrating multiple bioinformatics approaches, including WGCNA, machine learning algorithms, and molecular docking, we achieved a comprehensive understanding of the disease across the genomic, cellular, and molecular levels.

Regarding biological processes, the intersection genes were primarily involved in the homeostasis of the number of cells, myeloid cell differentiation, and myeloid cell homeostasis. Dysregulation of cell homeostasis is a hallmark of autoimmune diseases. In SLE, uncontrolled proliferation and activation of immune cells can result in autoantibody production and tissue damage (Herrada et al. 2019). Myeloid cells, including macrophages and dendritic cells, are integral to the immune response. Therefore, their abnormal differentiation and function may initiate autoimmunity (Liu et al. 2022; Arnaud et al. 2024). The involvement of these identified genes in myeloid cell functions indicates that they may be essential for dysregulated immune responses characteristic of SLE. Regarding cellular components, the genes were primarily associated with the cell cortex and basolateral plasma membrane. These components are critical for fundamental cellular processes such as cell signaling, adhesion, and motility. Alterations in their structure or function can impair intercellular communication and induce abnormal immune activation. Because such abnormal cell‐cell interactions and signaling dysregulation are considered central to SLE pathogenesis, these gene associations strongly implicate them in the underlying disease mechanisms.

Regarding molecular functions, the genes were enriched in activities, including structural elements of the cytoskeleton, aminoacyltransferase activity, and binding to 2 iron–2 sulfur clusters. The cytoskeleton is essential for preserving cell morphology and facilitating mobility; therefore, disruption of cytoskeletal integrity can impair immune cell function and is often associated with autoimmune diseases. Aminoacyltransferases, which are essential for protein synthesis, may lead to abnormal protein production when dysregulated, potentially affecting immune regulation. Furthermore, proteins that bind to iron‐sulfur clusters are essential for redox reactions and metabolic functions. Therefore, their dysfunction can impair cellular metabolism and promote inflammatory responses relevant to SLE pathology.

Pathway enrichment analysis indicated the significant enrichment of overlapping genes in pathways associated with malaria, bile secretion, and porphyrin metabolism. Although the association with the malaria pathway may seem unexpected, previous studies have proposed that immune activation mechanisms implicated in malaria infection may intersect with those in autoimmune conditions, such as SLE. This finding indicates a possible shared immunopathogenic landscape between infectious and autoimmune diseases, warranting further investigations (Bansal et al. 2017). For instance, both malaria and SLE involve immune activation and cytokine synthesis. The involvement of the bile secretion pathway may be associated with the liver's role in lipid and toxin metabolism (Pan et al. 2019). In SLE, liver function may be compromised, and alterations in bile secretion may contribute to the overall pathophysiology of the disease. Porphyrin metabolism is essential for heme synthesis, a constituent of several proteins, including hemoglobin and cytochrome enzymes. Consequently, dysregulation of porphyrin metabolism may result in porphyrin accumulation, leading to oxidative stress and tissue damage, both of which are features of SLE (Zhang et al. 2022). It should be objectively stated that the association between malaria, bile secretion pathways, and SLE in this study is currently based only on clues from enrichment analysis and indirect inferences from existing research. Their direct regulatory mechanisms have not yet been fully clarified and require further verification through subsequent functional experiments (such as gene silencing and in vitro cell model validation).

Our study introduced a novel approach by integrating multiple machine learning algorithms, including LASSO regression and RF algorithms, to identify key diagnostic biomarkers. LASSO regression identified eight potential diagnostic biomarkers, whereas RF selected ten diagnostic genes. The convergence of these two gene sets yielded four robust diagnostic biomarkers: SLC4A1, GATA1, DMTN, and SNCA. Subsequently, we developed an ANN diagnostic model based on the weighted contributions of these genes. The evaluation of this model via the ROC curve demonstrated its excellent diagnostic efficacy. The high AUC values for each of the four genes in the training and validation cohorts demonstrated that these genes have a strong discriminatory power for SLE diagnosis. Furthermore, forest plot analysis revealed that GATA1, DMTN, and SNCA were statistically significant risk factors for SLE, whereas SLC4A1 was a protective factor. These findings provide valuable diagnostic tools and offer critical insights into the role of these genes in SLE pathogenesis. The immune infiltration analysis, performed using the CIBERSORT algorithm, revealed significant differences in the infiltration levels of various immune cell populations between SLE and healthy control groups. The increased infiltration of CD8+ T cells, activated memory CD4+ T cells, and other immune cells in patients with SLE is consistent with the autoimmune nature of the disease. These immune cells are involved in the immunological response against self‐antigens, leading to tissue damage and inflammation. The correlation analysis between the key genes and immune cell populations offered further insights into the role of these genes in the immune response (Ohl and Tenbrock 2015; Choi et al. 2016).

For instance, GATA1 was significantly positively correlated with CD8+ T and resting memory CD4+ T cells. GATA1 is a transcription factor essential for the development and function of hematopoietic cells, including T cells (Takasaki and Chou 2024). Its positive correlation with T‐cell populations suggests its involvement in regulating T‐cell activation and differentiation in SLE (Wang et al. 2022). DMTN, SLC4A1, and SNCA were significantly correlated with several immune cell populations, highlighting their potential roles in immune‐mediated SLE pathogenesis. Findings from single‐cell RNA sequencing further reinforced this notion, demonstrating that all four essential genes were predominantly and highly expressed in T cells. Given that T cells are key regulators of immune function and that their dysregulation is a hallmark of SLE, the elevated expression of these genes in T cells indicates that they may contribute to abnormal T cell activation and function in this disease.

GSEA provided additional insights into the functional significance of these genes. Notably, diminished DMTN expression was enriched in immune‐related pathways, including antigen processing and presentation, autoimmune thyroid disease, cytosolic DNA sensing, natural killer cell‐mediated cytotoxicity, autophagy control, and RIG‐I‐like receptor signaling. These pathways are essential for maintaining immunological homeostasis and self‐tolerance. DMTN dysregulation may thus facilitate the disruption of self‐tolerance and the onset of autoimmunity in patients with SLE.

Conversely, high SNCA expression was correlated with pathways involved in drug metabolism via cytochrome P450, hematopoietic cell lineage, nitrogen metabolism, and porphyrin and chlorophyll metabolism. Although these pathways are not directly associated with immune regulation, their participation may reflect broader disturbances in cellular metabolism and hematopoietic function in patients with SLE, potentially affecting immune cell survival, differentiation, and systemic responses.

Developing a nomogram model for SLE diagnosis utilizing the key genes SLC4A1, GATA1, DMTN, and SNCA is a significant achievement. The nomogram is a graphical tool used to predict the probability of a patient with SLE based on the expression levels of these genes. The nomogram's strong performance, evidenced by the high agreement on the calibration curve and its exceptional predictive ability for SLE risk in the decision curve analysis, suggests significant potential for clinical adoption. It can provide clinicians with a more accurate and convenient method for diagnosing SLE, which is essential for early intervention and treatment. The systematic prediction of potential therapeutic interventions for SLE using the DSigDB database identified several compounds, with N‐acetyl‐L‐cysteine and iron emerging as the most statistically significant options. The molecular docking results revealed a stable interaction between N‐acetyl‐L‐cysteine and SNCA (binding energy: –4.6 kcal/mol). For comparison, the binding energy of belimumab to its target BAFF is –10.8 kcal/mol (measured by surface plasmon resonance) (Kardani et al. 2024), while hydroxychloroquine binds to TLR7/9 with energies between –6.1 and –7.3 kcal/mol (assessed using molecular dynamics simulations). Although N‐acetyl‐L‐cysteine exhibits a moderately lower affinity, its binding energy is comparable to other FDA‐approved small‐molecule drugs targeting PPIs (typical range: –4.0 to –7.0 kcal/mol). N‐acetyl‐L‐cysteine is a well‐known antioxidant and mucolytic agent. Regarding SLE, its antioxidant activities may mitigate oxidative stress, a significant factor in tissue damage and inflammation in the disease (Raghu et al. 2021). The stable interaction with SNCA, a protein associated with multiple cellular processes, indicates that N‐acetyl‐L‐cysteine may exert its therapeutic effects by regulating the function of SNCA and related pathways (Abbasifard et al. 2023; Perl 2013).

### 4.1 Limitations and Future Directions

This study has some limitations. First, this study was based on bioinformatics analysis of publicly available datasets, necessitating further experimental validation through in vitro and in vivo models to confirm the findings, as indicated in previous studies (Liu et al. 2025; Liu et al. 2024). Second, the candidate drug prediction was limited by the availability of data in the DSigDB database. Therefore, more comprehensive databases and experimental approaches are required to investigate a wider range of potential therapeutic agents. Third, the molecular docking simulations provided only an in silico prediction of the binding affinity between the drug and the target protein; consequently, additional experimental studies, including X‐ray crystallography or nuclear magnetic resonance spectroscopy, are required to precisely determine the binding mode and mechanism. Future studies should involve extensive clinical trials to confirm the diagnostic and prognostic value of the identified key genes and the nomogram model. Furthermore, investigating the molecular mechanisms governing the interactions between key genes and the immune system, as well as the development of targeted therapies based on identified candidate drugs, are important areas for future research. Integrating multi‐omics data, including genomics, transcriptomics, proteomics, and metabolomics, may offer a more comprehensive understanding of SLE pathogenesis and help identify new therapeutic targets.

## 5 Conclusion

Our study established SLC4A1, GATA1, DMTN, and SNCA as potential diagnostic biomarkers and highlighted N‐acetyl‐L‐cysteine as a novel therapeutic candidate. These insights not only advance our understanding of SLE pathogenesis but also pave the way for improved diagnosis and targeted therapies.

## Author Contributions

**Luofei Huang**: conceptualization, methodology, writing – original draft, visualization. **Quanzhi Lin**: conceptualization, data curation, writing – original draft. **Han Li**: formal analysis, investigation, writing – review and editing**. Jian Shi**: supervision, writing – review and editing, project administration. Additionally, all authors consented to the publication of this study.

## Funding

The authors have nothing to report.

## Conflicts of Interest

The authors declare no conflicts of interest.

## Supporting information




**Supplementary Information**: brb371098‐sup‐0001‐TableS1.csv

## Data Availability

The data utilized in this study can be accessed through the GEO database (https://www.ncbi.nlm.nih.gov/geo/).
